# *Bacillus coagulans* MF-06 alleviates intestinal mucosal barrier from damage in chicks infected with *Salmonella pullorum* via activating the Wnt/*β*-catenin pathway

**DOI:** 10.3389/fmicb.2024.1492035

**Published:** 2024-11-29

**Authors:** Li Ma, Guangming Tian, Yuejin Pu, Xuguang Qin, Yinghu Zhang, Haojie Wang, Lei You, Gaofeng Zhang, Chun Fang, Xiongyan Liang, Hongbo Wei, Lei Tan, Liren Jiang

**Affiliations:** ^1^College of Animal Science and Technology, Yangtze University, Jingzhou, China; ^2^State Key Laboratory of Biocatalysis and Enzyme Engineering, School of Life Sciences, Hubei University, Wuhan, China; ^3^Hubei Provincial Livestock Technology Extension Center, Wuhan, Hubei, China; ^4^Animal Disease Prevention and Control Center of Rizhao City, Shandong, China; ^5^Yiling District Agricultural Product Quality and Safety Service Center, Yichang, Hubei, China

**Keywords:** *Bacillus coagulans*, *Salmonella pullorum*, chick, intestinal mucosal barrier, Wnt/*β*-catenin pathway, gut microbiota

## Abstract

**Introduction:**

This study aimed to assess the protective efficacy of *Bacillus coagulans* MF-06 as a potential alternative to antibiotics in mitigating intestinal mucosal damage in chicks infected with *Salmonella pullorum*.

**Methods:**

A total of 150 one-day-old SPF chicks were selected and randomly divided into five groups: control group (CK), probiotics group (EM), probiotics treatment group (PT), antibiotic treatment group (AT), *Salmonella pullorum* group (SI), CK, AT and SI groups were fed a basal diet, EM and PT groups were fed a basal diet supplemented with 1.0 × 10^8^ CFU/g *Bacillus coagulans*; PT, AT and SI groups were gavaged with 1.0 × 10^9^ CFU/0.5 mL *Salmonella pullorum* at 7 days of age; AT group were fed with 0.375 g/kg neomycin sulfate in the basal diet from days 7–14.

**Results:**

Subsequently, the study evaluated alterations in growth performance, the integrity of the intestinal mucosal barrier, cytokines associated with the Wnt/*β*-catenin signaling pathway, and gut microbiota composition. The results revealed that the administration of *Bacillus coagulans* MF-06 significantly reduced the feed conversion ratio of chicks (*p* < 0.05), and significantly increased the average daily weight gain and average daily feed intake in chicks challenged with *Salmonella Pullorum* (*p* < 0.05). Furthermore, *Bacillus coagulans* MF-06 treatment diminished the presence of *Salmonella pullorum* colonies in the intestinal tract. Additionally, the administration of *Bacillus coagulans* MF-06 restored levels of (Diamine oxidase) DAO and (D-lactic acid) D-LA levels, as well as the levels of tight junction protein, including *TJP1*, *CLDN1*, *CLDN2*, *Occludin*, and *MUC2* (*p* < 0.05). The study noted a significant decrease in cell apoptosis (*p* < 0.05) and a significant increase in the expression of Proliferating Cell Nuclear Antigen (PCNA) and v-myc avian myelocytomatosis viral oncogene homolog (C-MYC) (*p* < 0.05), which activated the Wnt/*β*-catenin signaling pathway. Analysis through 16S rRNA sequencing revealed that the intake of *Bacillus coagulans* MF-06 led to a significant decrease in the relative abundance of *Lachnoclostridium*, *Shuttleworthia*, and *unidentified-Eggerthellaceae* (*p* < 0.05).

**Discussion:**

Collectively, the *Bacillus coagulans* MF-06 may provide a protective effect against *Salmonella pullorum* infection in chicks by enhancing growth performance, strengthening the integrity of the intestinal mucosal barrier, and stabilizing the gut microbiota.

## 1 Introduction

*Salmonella pullorum* (*S. pullorum*) is the causative agent of Pullorum disease (PD), which primarily affects chicks, turkeys, and other domestic poultry at 1–2 weeks of age. Clinical manifestations of chicks include anorexia, white diarrhea, weakness, and increased mortality, causing severe economic losses to the poultry industry in developing countries (Shivaprasad, 2000; Barrow and Neto, 2011; Jiaqi et al., 2022; Shen et al., 2022). *S. pullorum* is transmitted by the vertical and horizontal routes, the vertical route facilitates the transmission of *S. pullorum* from hens to chicks via eggs, while the horizontal route is considered the main transmission strategy of this pathogen in which chicks are infected by ingesting the contaminated feed and water (Cheng et al., 2020). For the horizontal route of *S. pullorum*, it initially colonization and attachment to the intestinal mucosal epithelium, causing severe damage to the intestinal function and microbiota of chicks, and subsequently spreads to host tissues and organs, weakening immunity, causing sepsis, and impairing immune responses. Therefore, the first step of *S. pullorum* infection is invasion of the intestinal mucosal epithelium (Wang et al., 2019; Wang et al., 2021b; Zou et al., 2024).

The production of various epithelial cells driven by ISCs is integral to the maintenance of the intestinal epithelial barrier, and the proliferation and apoptosis of ISCs are essential for maintaining intestinal homeostasis (Ding et al., 2024). Various signaling pathways, including the Wnt/*β*-catenin pathway, exert significant influence on intestinal homeostasis. Wnt/*β*-catenin pathway-related proteins are abundant in the gut microenvironment, and they play a crucial role in intestinal epithelial renewal (Hao et al., 2024). The Wnt ligand is released by Paneth, stromal, and endothelial cells located below the epithelial surface, including macrophages. Upon release, Wnt activates low-density lipoprotein receptor-associated protein 5/6 (LRP5/6) and Frizzled co-receptor, promoting β-catenin nuclear translocation, where it interacts with transcription factor 4 (TCF4) to maintain stem cell and epithelial cell proliferation and differentiation (Lee et al., 2018). Therefore, Enhancement of the intestinal mucosal barrier is essential for defense against *S. pullorum* in chicks (Deng et al., 2021; Liangyu et al., 2022).

Traditionally, antibiotic therapy has been considered the main prevention and treatment strategy for *S. pullorum*, but the persistent and widespread use of antibiotics can lead to the emergence of antibiotic-resistant strains, drug residues, and microbial dysbiosis in animals, posing a potential threat to human health (Song et al., 2020). Since 2006, the European Union (EU) has banned the application of antimicrobial growth promoters in animal feed and water (Martin et al., 2015). Subsequently, China and the United States have also banned the use of antibiotics as feed additives (Wang et al., 2022; Attia et al., 2023). Therefore, safer and more effective alternatives to antibiotics need to be developed or discovered.

It is reported that probiotics can enhance food safety and promote the health of animal gastrointestinal tracts by generating organic acids, stimulating the host immune response, and producing antimicrobial substances (Wang et al., 2021a). Among the various probiotics, *Bacillus coagulans* (*B. coagulans*), as a facultative anaerobic, Gram-positive, and non-pathogenic bacterium, can form spores and produce lactic acid (Hou et al., 2023). Spore-forming *B. coagulans* exhibits strong resistance to extreme environmental conditions, which allows it to adapt to the acidic and hypoxic environment of the gastrointestinal tract and survive in the intestine, where it exerts the functions of lactic acid bacteria, *B. coagulans* can also diminish intestinal pH, facilitate the proliferation of advantageous bacteria, and impede the growth of detrimental microorganisms, which has potential application value of alternative antibiotics (Hung et al., 2012; Zhang et al., 2021). At present, *B. coagulans* is widely used in aquaculture, livestock, and poultry (Zhou et al., 2020; Liu et al., 2022; Hu et al., 2024). For example, Dietary *B. coagulans* supplementation attenuated inflammation by modulating Th cell function and T cell responses, and restored intestinal microflora by enriching beneficial bacteria and suppressing harmful flora (Guo et al., 2021); Addition of 1.0 × 10^8^ CFU/mL *B. coagulans* to the water can improves growth performance, enhances intestinal innate immunity and improves intestinal microbial communities of broilers (Liu et al., 2022); Addition of 2.0 × 10^8^ CFU/g *B. coagulans* to the feed could considerably improve the growth performance of broilers (Hou et al., 2023); Dietary supplementation of *B. coagulans* in piglets infected by Enterotoxigenic *Escherichia coli* (ETEC) K88 can prevent the decrease in average daily feed intake by alleviating intestinal damage and modulating the gut microbiota (Zhang et al., 2023). However, there have been few reports about the effects of avian autochthonous probiotics on the intestinal mucosal barrier and gut microbiota of chicks infected with *S. pullorum*. In view of those, this study explored the effects of *B. coagulans* MF-06 on the growth performance, intestinal mucosal barrier, and gut microbiota of chicks infected with *S. pullorum*.

## 2 Materials and methods

### 2.1 Ethics statement

The experiments were approved by the Institutional Animal Care and Use Committee of Yangtze University (Approval No. 202401003).

### 2.2 Preparation of probiotics and source of neomycin sulfate

*Bacillus* *coagulans* strain MF-06 was previously isolated and identified in our laboratory. Briefly, the strain was isolated from the intestinal contents of chicks and then identified through physiological, biochemical, and molecular biological analyses. The dominant *B. coagulans* strains MF-06 were selected based on the preliminary results of physiological, biochemical, and *in vitro* antibacterial experiments (Lee et al., 2014). The isolated probiotics were prepared into freeze-dried powder, which was modified according to Chen et al. (2020) the research method. The glycerin storage tubes containing strains were inoculated with 2% inoculate into YPD medium and activated for three generations. Strains were activated with 2% inoculum to 100 mL YPD medium at 37°C for 16 h. Then, 2% of the activated strains were inoculated into 2.5 L YPD medium and incubated in a 5 L bioreactor (FR-P series; Shanghai Jichen Biotechnology Co., LTD., Shanghai) in the high-density fermentation, 37°C for 16 h. The bacterial sludge was centrifuged at 4°C for 20 min at 8000 rpm and collected. The bacteria pellet was resuspended in 250 mL of sterilized degreased milk powder protectant, mixed, and frozen at −80°C for 12—14 h. The frozen samples were then freeze-dried under vacuum (ZLGJ-18 multi-manifold model, Huachen Instrument Co., LTD., Zhengzhou, China) at −50°C for 48 h. This freeze-dried powder was the final probiotic. Lyophilized powder was prepared and viable counts per gram of *B. coagulans* were determined by the plate coating method. The samples were then stored at 4°C until use. Neomycin sulfate-soluble powder was purchased from Inner Mongolia Huatian Pharmaceutical Co. Ltd. (Inner Mongolia, China).

### 2.3 Culture of *Salmonella pullorum*

The *S. pullorum* strain (CVCC No. 534) was obtained from the China Veterinary Microbial Strain Preservation and Management Center in Beijing, China. The species of bacteria were preserved in 20% glycerol at −80°C. Bacterial viability was measured by the plate smear method, and the corresponding Optical density (OD) values were measured to plot the growth curve of *S. pullorum*. Bacterial suspensions were prepared after determined the concentrations.

### 2.4 Animal experiments

A total of 150 one-day-old SPF chicks (Jinghong No.1) were Hubei Yukou Poultry Industry Co., Ltd., Hubei. They selected and randomly divided into five groups: CK group: control group, which was fed with basic diet with no administration of antibiotics or probiotics. EM group: probiotics group, whose basal diet was added with 1.0 × 10^8^ CFU/g *B. coagulans* MF-06; PT group: probiotics treatment group, whose basal diet was added with 1.0 × 10^8^ CFU/g *B. coagulans* MF-06 from days 1 to 14, and when chicks were seven-day-old, 1.0 × 10^9^ CFU/0.5 mL of *S. pullorum* was administered by gavage; AT group: antibiotic treatment group, for whom basal diet was provided for the first 7 days, and when chicks were seven-day-old, they administered with 1.0 × 10^9^ CFU/0.5 mL of *S. pullorum* by gavage, and the basal diet supplemented with 0.375 g/kg neomycin sulfate from days 7 to 14. SI group: *S. pullorum* infection group, which was fed with basic diet, and when chicks were seven-day-old, they were administered with 1.0 × 10^9^ CFU/0.5 mL of *S. pullorum* by gavage (Deng et al., 2021); Each group comprised six replicates, with each replicate consisting of five chicks. The added dose of *B. coagulans* was obtained from a preliminary dose exploration experiment. The trial design is shown in Supplementary Figure S1, the chicks in the CK and EM groups were accommodated in an independent house in case of cross-contamination and humidity, and chicks in each group were housed in wire cages (110 × 80 × 45 cm). All chicks tested negative for pathogens before the experiment and the chicken house was thoroughly sterilized before the experiment. During the 14-day experiment, all chicks were allowed unrestricted access to water and fed with a starter feed devoid of antibiotics. All feeds were ground into powders and stored in a low-temperature dry environment, the basal diet composition and basic feed composition are presented in Table 1. At 9 am, the time of the first feeding and water change, chickens were observed for any abnormalities. The second feeding and water change was at 3:00 pm, when the chickens were observed again. The amount of feed remaining was recorded daily and the enclosure was cleaned regularly. The same rearing conditions were used, and the chick chambers were maintained at 34–36°C and a 60% relative humidity from day 1 to day 3. The temperature was decreased by 1°C each day from day 4 to day 14 and maintained at 31–33°C. The chambers were illuminated for 24 h on days 1 to day 3, for 22 h on days 4 to day 7, and for 20 h on days 8 to day 14. The growth status, body weight, survival rate, and clinical symptoms were recorded daily.

**Table 1:** Basal diet composition and nutrition content of the feed for chicks in this study.

Diet composition	Content (%)	Nutritional level	Content (%)
Corn	53.75	ME(MJ/kg)	11.91
Flour	10.00	Crude protein^2^^,^^3^	19.65
Soya bean meal	26.70	Calcium^2^^,^^3^	0.98
Imported fish meal	2.50	Available phosphorus^2^^,^^3^	0.45
Bran	2.00	Digestible lysine	1.05
Soya-bean oil	1.00	Digestible methionine	0.50
Mountain flour	1.10	Digestible threonine	0.73
Calcium hydrogen phosphate	1.20		
L-lysine hydrochloride	0.20		
DL-Methionine	0.15		
L Threonine	0.10		
Salt	0.25		
60 Choline chloride	0.05		
Premix^1^	1.00		
Total	100.00		

^1^The premix provides the following per kilogram of diet: vitamin A, 10,000 IU/kg; vitamin D, 4500 IU/kg; vitamin E, 40 mg/kg; vitamin K_3_, 3.5 mg/kg; vitamin B_1_, 3 mg/kg; vitamin B_2_, 7 mg/kg; vitamin B_6_, 5.5 m/kg; vitamin B_12_, 0.03 mg/kg; pantothenic acid, 13 mg/kg; niacin, 40 mg/kg; folic acid, 2.1 mg/kg; biotin, 0.25 mg/kg; Copper, 10 mg/kg; iron, 60 mg/kg; zinc, 80 mg/kg; manganese, 100 mg/kg; iodine, 1.4 mg/kg; selenium, 0.4 mg/kg. ^2^Crude protein, calcium, and phosphorus were actually measured values. Other nutrient levels were calculated using the China Feed Database (https://www.chinafeeddata.org.cn/). ^3^Crude protein was analyzed using the Kjeldahl method, in accordance with the Chinese national standard (GB/T 6432–2018). Calcium levels were measured following the Chinese national standard (GB/T 6436–2018). Phosphorus analysis was performed using a 731 N visible spectrophotometer (Shanghai Youke Instrument Co., Lid, Shanghai, China) following the Chinese national standard (GB/T 6437–2018).

### 2.5 Sample collection

On the 14th day, two chicks were randomly selected from each replicate group for a total of six chicks per group. Two mL of blood sample was collected from the vein under the wing and stored in procoagulant tubes, followed by incubation at room temperature for 2 h and centrifugation at 3,000 *g* and 4°C for 15 min for separation of the serum, which was then collected and stored at −80°C for subsequent analysis. The chicks were euthanized under anesthesia by cervical dislocation, and then sample was aseptically collected from the duodenum, jejunum, ileum, and cecum and stored at 4°C for quantifying the bacterial load. To perform 16S rRNA sequencing analysis, 0.5 g of the remaining cecal content was collected, frozen in liquid nitrogen, and stored at −80°C. The jejunum (50 mg) was aseptically collected and immersed in an RNA sample preservation solution, flash-frozen with liquid nitrogen, and stored at −80°C for total RNA isolation. Additionally, Midsections (3—5 cm) of the jejunum were collected and fixed in a 4% paraformaldehyde solution for subsequent immunohistochemical analysis.

### 2.6 Serum biochemical indices

The D-lactic acid (D-LA) level in the serum was assessed with a commercially available ELISA kit (Shanghai Enzyme Linked Biology Co., Ltd., Shanghai, China); The diamine oxidase (DAO) level in the serum was measured with a commercially available kit (Nanjing Jiancheng Bioengineering Institute, Nanjing, China).

### 2.7 Colony counts of *Salmonella pullorum*

The bacterial load in tissues was determined according to the previous method with some modifications (Li et al., 2024), the tissue specimens of duodenum, jejunum, ileum, and cecum (100 mg) after sterile collection were placed in 2 mL sterile centrifuge tube. Subsequently, 900 μL of PBS buffer was added, and the mixture was thoroughly homogenized. Following gradient dilution (10^−2^–10^−8^), all samples were inoculated on bismuth sulfite agar; the process six experiments were repeated, with each replicate conducted three times, and then the samples were cultured at 37°C for 18–24 h. The morphological characteristics of the colonies were used to quantify *S. pullorum* in the intestinal tissue. *S. pullorum* counts, expressed as logarithmic transformation (log_10_), were reported as colony-forming units per 0.1 g (CFU/0.1 g).

### 2.8 Intestinal length index

Before the chicks were dissected, body weight was recorded, duodenum, jejunum, and ileum were isolated, and the lengths of the corresponding parts were measured. Three parallel biological replicates were used for all procedures and measurements.

### 2.9 Immunohistochemical analysis

Small segments of mid-jejunum were fixed with 4% paraformaldehyde solution and dehydrated through an ascending ethanol gradient. The segments were cleaned with xylene and embedded in paraffin. Next, the samples were sectioned at 5 μm for immunohistochemical analysis of Proliferating Cell Nuclear Antigen (PCNA) and v-myc avian myelocytomatosis viral oncogene homolog (C-MYC), the immunohistochemistry was conducted following the method by previous research (Shen et al., 2024). The sections were dewaxed with xylene, anhydrous ethanol, distilled water and incubated with 3% H_2_O_2_ for 20 min at room temperature (25 ± 2°C) to restrict the endogenous peroxidase activity. Then, the citric acid buffer (G1202 Servicbio Co., Ltd., Wuhan, China) was used to restore antigen activity for 10 min. Next, the sections were immersed in 3% Bovine serum BSA (GC305010, Servicbio Co., Ltd., Wuhan, China) for 30 min at room temperature. Gently shake off the sealing solution, add PBS to the section in 1:500 proportion of primary antibody (Anti -PCNA Rabbit pAb and Anti -C-MYC Rabbit pAb), and the section is placed flat in a wet box at 4°C for overnight incubation. The slide was placed in PBS (pH 7.4) and washed by shaking on the decolorizing shaker for three times, 5 min each time. Next, the sections were incubated with biotinylated goat anti-rabbit antibody (1:200, GB23303 Servicbio Co., Ltd., Wuhan, China) at room temperature for 50 min, and washed with PBS (pH 7.4) for three times. After the sections were slightly dried, the freshly prepared DAB color developing solution (G1212, Servicbio Co., Ltd., Wuhan, China) was added into the circle, the color developing time was controlled under the microscope. The positive color was brown and yellow, and the sections were rinsed with tap water to terminate the color development. Next, hematoxylin restaining for about 3 min, washing with tap water, hematoxylin differentiation solution for a few seconds, rinse with tap water, hematoxylin return to blue solution, and rinse with running water. Then were conventionally dehydrated to transparency and sealed using neutral balsam. Four sections were randomly selected for each group and chose three different regions of each section. The average optical density of immune positive substances in the intestine was measured using ImageJ software (version 1.8).

### 2.10 RNA isolation and quantitative real-time PCR

Total RNA was extracted from jejunum samples using the TRIzol™ Reagent Kit (Thermo Fisher Scientific, Waltham, Massachusetts, USA) according to the manufacturer’s protocol, and the RNA concentration was measured using a microspectrophotometer (nanodrop2000, Thermo Fisher Scientific, Massachusetts). cDNA was synthesized from the RNA using the Color Reverse Transcription Kit with gDNA Remover (EZBioscience, USA). All gene expression levels were normalized with *β*-actin as the internal standard. PCR primer sequences (Table 2) were designed and synthesized by Shanghai SANGON bio (Shanghai, China). SYBR Green fast qPCR mix (RK21203; Abclonal, Wuhan, China) was used with the following program: 95°C for 3 min, 30 cycles of 95°C for 5 s, 60°C for 30 s. This experiment was performed in six biological replicates with three replicates each. The quantitative RT-PCR (RT-qPCR) assay was performed to analyze the relative mRNA levels of the targeted genes using the 2^-△△CT^ method as previously described (Livak and Schmittgen, 2001).

**Table 2:** Primer sequences for in real-time quantitative qRT-PCR assays.

Gene name	Accession NO.	Primer sequence (5′ − 3′)	Product (bp)
TJP1	XM_021098856.1	F: CAGCCGAAAGCAGAAGCATCAC R: AAGAGTTGTAAGGAGCAGCAGTAGG	151
CLDN1	NM_001013611.2	F: ACCAGGTGAAGAAGATGCGGATG R: ACGGGTGTGAAAGGGTCATAGAAG	133
CLDN2	NM_001277622.1	F: AAGCATCGTGACAGCCGTGAG R: GCAAGGGAGGAGACAGCACAG	168
Occludin	NM_205128.1	F: CTACGGCAGCACCTACCTCAAC R: GGCAGAGCAGGATGACGATGAG	107
MUC2	XM_040701656.2	F: CGACAATTACCACCATACCAACACC R: CACACCAGGAACAGTAGGAGTAGC	188
Wnt3	XM_046904315.1	F: CCATCTGCGGCTGTGACTCC R: CCTCGTTGTTGTGTCTGTTCATGG	168
TCF4	XM_046905872.1	F: TTCTCCAGATCACACCAACACCAG R: GCGTCGTCCAACCTCTCCAAG	192
C-MYC	NM_001030952.2	F: AGCAGCGACTCGGAAGAAGAAC R: CGTAGTTGTGTTGGTGGATGTTGAC	184
CTNNB	NM_205081.3	F: TCACCTCACCAGCAGACATCAG R: GCGAATCAACCCAACAGTAGCC	139
BMI1	NM_001007988.3	F: CCCGCAGTTCCCACACATCTC R: TTCCGTGCTCTGTTGGTGAAGG	93
LGR5	XM_425441.7	F: TCCTACTGAAGCACTCCAGAACTTG R: CGTCCAGCCACAGGTGTCTAAG	125
BAX	FJ977571.1	F: CTACTCCTCATACGCTGTCTTCCTG R: TAGTCTGGTACATGCTGGCAATGG	155
BCL2	NM_205339.3	F: CAGGACAACGGAGGATGGGATG R: CACCAGAACCAGGCTCAGGATG	111
CASP8	NM_204592.4	F: CTTCCTCTTGGGCATGGCTACC R: GCTTGTCAATCTTGCTGCTCACC	176
CASP3	NM_204725.2	F: AGCAGACAGTGGACCAGATGAAAC R: TGGCGTGTTCCTTCAGCATCC	161
β-Actin	NM_205518.2	F: ATGGCTCCGGTATGTGCAAG R: CAACCATCACACCCTGATGTC	102

TJP1, tight junction protein 1; CLDN1, claudin 1; CLDN2, claudin 2; OCLD, Occludin; MUC2, mucin 2; Wnt3, Wnt family member 3; TCF4, transcription factor 4; C-MYC, v-myc avian myelocytomatosis viral oncogene homolog; CTNNB1, catenin beta; BMI1, BMI proto-oncogene, polycomb ring finger; LGR5, leucine rich repeat containing G protein-coupled receptor 5; BAX, bcl2-associated X protein b; BCL2, apoptosis regulator; CASP8, caspase 8; CASP3, caspase 3; β-Actin, actin, beta.

### 2.11 Microbial 16S rRNA gene sequencing

For each group, samples of cecal content were randomly selected for full-length 16S rRNA sequencing. Total genome DNA from samples was extracted using CTAB method. DNA concentration and purity was monitored on 1% agarose gels. According to the concentration, DNA was diluted to 1 ng/L using sterile water (Jiali et al., 2024). 16S rRNA genes of regions (V3-V4/16S) were amplified used specific primer (F: 5′-CCTAYGGGRBGCASCAG-3′, R: 5’-GGACTACNNGGGTATCTAAT-3′). All PCR reactions were carried out with 15 L of Phusion® High-Fidelity PCR Master Mix (New England Biolabs); 2 M of forward and reverse primers, and about 10 ng template DNA. Thermal cycling consisted of initial denaturation at 98°C for 1 min, followed by 30 cycles of denaturation at 98°C for 10 s, annealing at 50°C for 30 s, and elongation at 72°C for 30 s. Finally, 72°C for 5 min. Mix same volume of 1X loading buffer (contained SYB green) with PCR products and operate electrophoresis on 2% agarose gel for detection. PCR products was mixed in equidensity ratios. Then, mixture PCR products was purified with Qiagen Gel Extraction Kit (Qiagen, Germany). Then, Sequencing libraries were generated using TruSeq® DNA PCR-Free Sample Preparation Kit (Illumina, USA) following manufacturer’s recommendations and index codes were added. The library quality was assessed on the Qubit@ 2.0 Fluorometer (Thermo Scientific) and Agilent Bioanalyzer 2,100 system. At last, the library was sequenced on an Illumina NovaSeq platform and 250 bp paired-end reads were generated.

### 2.12 Diversity analysis

Paired-end reads was assigned to samples based on their unique barcode and truncated by cutting off the barcode and primer sequence. Quality filtering on the raw tags were performed under specific filtering conditions to obtain the high-quality clean tags according to the fast (v0.22.0).^1^ Paired-end reads were merged using FLASH (v1.2.11),^2^ The tags were compared with the reference database^3^ using UCHIME Algorithm^4^ to detect chimera sequences, and then the chimera sequences were removed. Then the Effective Tags finally obtained. Sequences analysis was performed by Uparse software (Uparse v7.0.1001).^5^ Sequences with 97% similarity were assigned to the same OTUs. Representative sequence for each OTU was screened for further annotation. Amplicon sequence variant (ASV) was analyzed by Deblur, which uses error profiles to obtain putative error-free sequences from Illumina MiSeq and HiSeq sequencing platforms. For each representative sequence, the Silva Database^6^ was used based on Mothur algorithm to annotate taxonomic information. OTUs abundance information were normalized using a standard of sequence number corresponding to the sample with the least sequences. Alpha diversity is applied in analyzing complexity of species diversity for a sample through 6 indices, including Observed-species, Chao1, Shannon, Simpson, ACE, Good-coverage. All these indices in our samples were calculated with QIIME and displayed with R software (Version 4.1.2). To further investigate the differences in community structure and variations in species composition among grouped samples, we performed Student *t*-tests and LEfSe analysis.

### 2.13 Statistical analysis

In this study, Kruskal-Wallis test was used to compare the differences of different microbial communities in each group, Because the data did not conform to the assumption of normal distribution, we chose to use nonparametric test methods to analyze differences in microbial diversity or abundance between groups. If the test results showed a significant difference (*p* < 0.05), a further Dunn’s multiple comparison test was performed to determine which groups were significantly different. Growth performance was analyzed by a Mixed Effects Model using SPSS software (version 25.0, SPSS Inc., Chicago, IL, USA), and other data, such as bacterial load, serum indicators and gene expression, were analyzed by one-way ANOVA significant differences between means were compared using Duncan’s multiple range test. Statistical significance of differences was expressed by the presence of a-d letters within a column; different letters within a column signify a significant difference between means (*p* < 0.05). Data visualization was achieved using GraphPad Prism 9.0 (GraphPad Inc., La Jolla, CA), and the ImageJ software (version 1.8) was employed to measure the data. All experimental data have at least three independent replicates, and the results are expressed as mean ± standard deviation (SD).

## 3 Result

### 3.1 Growth performance

Table 3 presents the impact of *B. coagulans* MF-06 on the growth performance of chickens infected with *S. pullorum*. On day 7, the body weight of the EM group (62.85 g) was significantly greater than that of the other groups (*p* < 0.05), while no significant differences were observed among the remaining four groups (*p* > 0.05). By day 14, the body weight of the SI group had decreased by 5.39% in comparison to the CK group (*p* < 0.05), the body weights of the PT and AT groups increased by 4.75 and 5.68%, respectively, when compared to the SI group (*p* < 0.05), the EM group exhibited the highest body weight at 126.50 g on day 14. During the experimental period (1–14 days), the SI group experienced reductions in ADG and ADFI of 7.30 and 2.6%, respectively, compared to the CK group, with an increase in FCR of 4.73% (*p* < 0.05). When compared to the SI group, the ADG in the PT and AT groups increased by 6.50 and 7.30%, respectively (*p* < 0.05), while ADFI increased by 2.54 and 3.20% (*p* < 0.05), additionally, FCR decreased by 3.51 and 4.02% in the PT and AT groups, respectively (*p* < 0.05). The average daily gain (ADG) in the EM group (6.70 g) was significantly higher than that of the other groups (*p* < 0.05), while the feed conversion ratio (FCR) was lower than that of the other groups (*p* < 0.05).

**Table 3:** Body weight (BW), average daily gain (ADG), average daily feed intake (ADFI), and feed conversion ratio (FCR) under different treatments.^1^

Item	CK	EM	PT	AT	SI	*p*-value
BW day 1 (g)	32.62 ± 0.83	32.76 ± 0.34	32.68 ± 0.46	32.99 ± 0.50	32.57 ± 0.15	0.642
BW day 7 (g)	60.09 ± 0.73^b^	62.85 ± 0.99^a^	60.14 ± 0.96^b^	60.89 ± 0.90^b^	60.37 ± 1.00^b^	<0.001
BW day 14 (g)	122.66 ± 3.23^b^	126.50 ± 2.45^a^	121.56 ± 1.62^b^	122.64 ± 2.04^b^	116.04 ± 1.67^c^	<0.001
ADG day 1–14 (g)	6.43 ± 0.24^b^	6.70 ± 0.18^a^	6.35 ± 0.10^b^	6.40 ± 0.16^b^	5.96 ± 0.12^c^	<0.001
ADFI day 1–14 (g)	12.19 ± 0.15^a^	12.27 ± 0.25^a^	12.18 ± 0.18^a^	12.25 ± 0.36^a^	11.87 ± 0.03^b^	<0.001
FCR day 1–14	1.90 ± 0.06^bc^	1.83 ± 0.04^c^	1.92 ± 0.03^b^	1.91 ± 0.05^b^	1.99 ± 0.07^a^	<0.001

CK, control group; EM, *B. coagulans* group; PT, probiotics treatment group; AT, antibiotic treatment group; SI, *S. pullorum* infection group. ^1^Values are presented as mean ± SD, *n* = 6. ^a, b, c^ Significantly different means are indicated by distinct superscripts within the same row (*p* < 0.05).

### 3.2 Colonization of *Salmonella pullorum* in intestinal tissues

**Figure 1:**
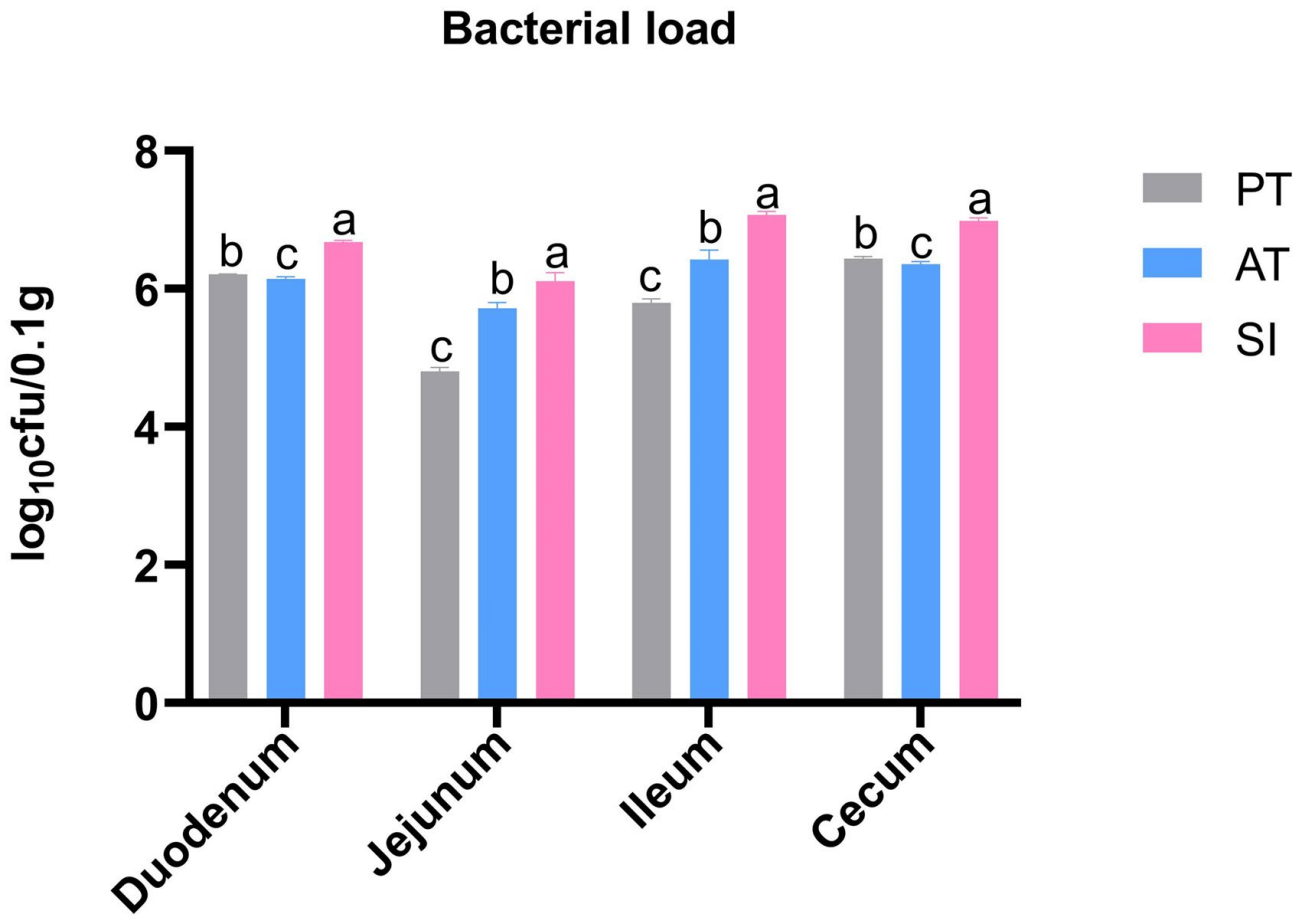
Logarithmic colony counts of *S. pullorum* in the duodenum, jejunum, ileum, and cecum of various treatment groups. Different lowercase letters indicate significant differences between groups. Data are presented as the mean ± SD, *n* = 6. CK, control group; EM, *B. coagulans* group; PT, probiotics treatment group; AT, antibiotic treatment group; SI, *S. pullorum* infection group.

The number of *S. pullorum* colonizing the duodenum, jejunum, ileum, and cecum in each group is shown in Figure 1. *S. pullorum* was not detected in the tissues from the CK and EM groups. The colonization levels in the duodenum, jejunum, ileum, and cecum of the SI group were 4.76 × 10^6^, 1.33 × 10^6^, 1.18 × 10^7^, and 6.73 × 10^6^ CFU/0.1 g, respectively, which were higher than those recorded in other groups (*p* < 0.05). For the PT and AT groups, the colonization number of *S. pullorum* was 1.62 × 10^6^ and 1.40 × 10^6^ CFU/0.1 g in duodenum, 6.41 × 10^4^ and 5.25 × 10^5^ CFU/0.1 g in jejunum, 6.31 × 10^5^ and 2.76 × 10^6^ CFU/0.1 g in ileum, and 2.72 × 10^6^ and 2.28 × 10^6^ CFU/0.1 g in cecum, respectively. These values were lower than those found in the corresponding parts of the SI group (*p* < 0.05).

### 3.3 Intestinal length index

The effects of *B. coagulans* MF-06 on intestinal index of chicks infected with *S. pullorum* are shown in Table 4. There was no significant difference in duodenal length index among the groups (*p* > 0.05). The jejunum length index was similar among the SI, PT, and AT groups (21.9, 20.89, 20.87%), and the values were significantly lower than that of the CK group (23.9%) (*p* < 0.05). The ileum length index in the SI group (17.29%) was lower than that in the CK group (22.51%) and PT group (20.54%) (*p* < 0.05), whereas showed no significant difference from that in the AT group (17.82%) (*p* > 0.05).

**Table 4:** Changes in the intestinal length index of different treatment groups.^1^

Item	CK	EM	PT	AT	SI	*p*-value
Duodenum (cm)	10.89 ± 0.31^b^	11.64 ± 0.15^a^	11.70 ± 0.35^a^	11.66 ± 0.49^a^	11.54 ± 0.39^ab^	0.088
Jejunum (cm)	23.90 ± 0.75^b^	23.61 ± 0.27^b^	20.89 ± 0.71^c^	20.87 ± 0.70^c^	21.90 ± 0.79^c^	<0.001
Ileum (cm)	22.51 ± 0.09^a^	20.89 ± 0.19^b^	20.54 ± 0.66^b^	17.82 ± 0.39^c^	17.29 ± 0.38^c^	<0.001

CK, control group; EM, *B. coagulans* group; PT, probiotics treatment group; AT, antibiotic treatment group; SI, *Salmonella pullorum* infection group. ^1^Values are presented as mean ± SD, *n* = 6. ^a, b, c^ Significantly different means are indicated by distinct superscripts within the same row (*p* < 0.05).

### 3.4 Detection of genes associated with intestinal barrier function

**Figure 2:**
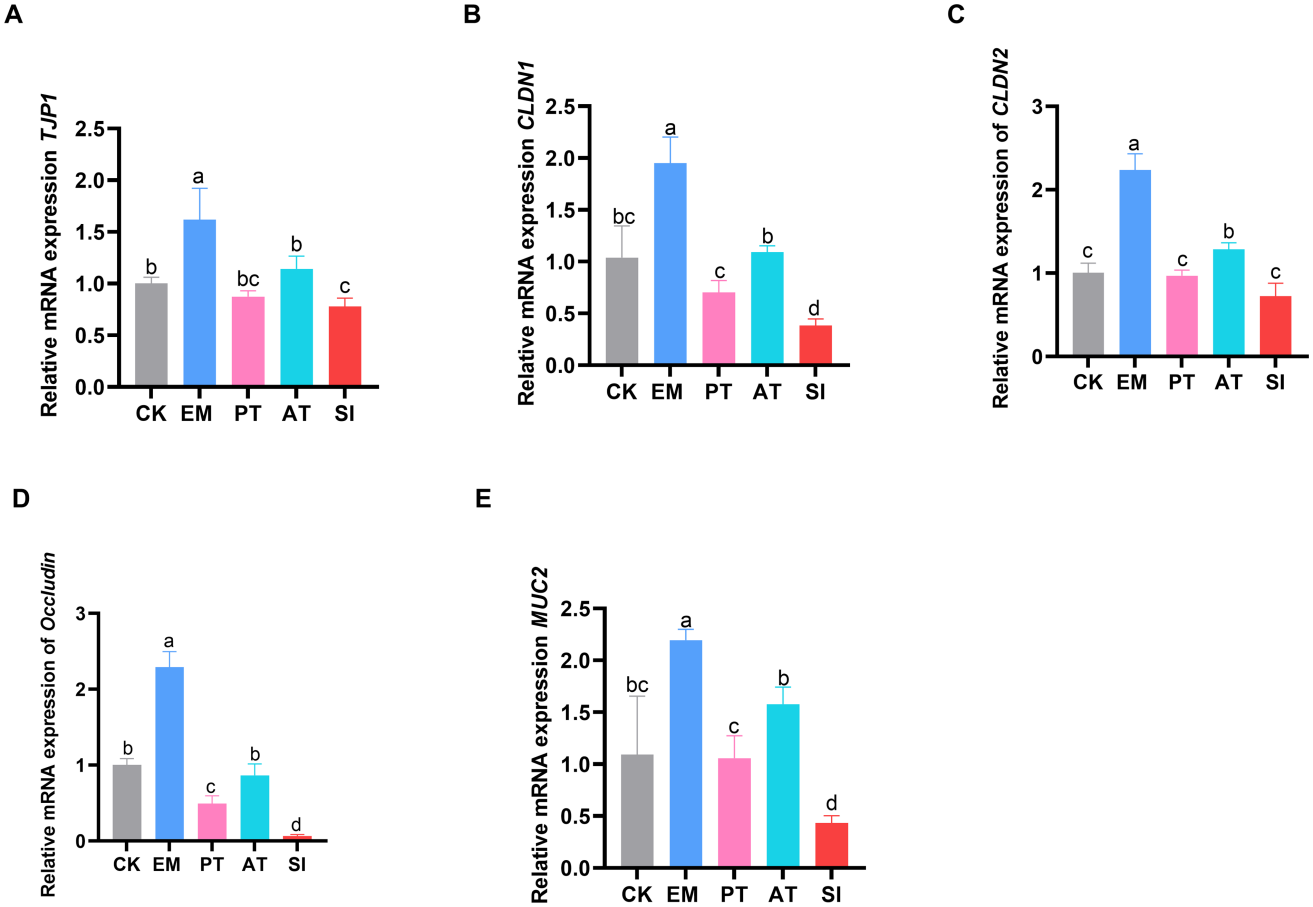
Expression of jejunal tight junction-related genes. **(A)** Relative expression of the mRNAs of *TJP1*, **(B)** *CLDN1*, **(C)** *CLDN2*, **(D)** *Occludin*, and **(E)** *MUC2*. Different lowercase letters indicate differences between groups. Values are presented as the mean ± SD, *n* = 6. CK, control group; EM, *B. coagulans* group; PT, probiotics treatment group; AT, antibiotic treatment group; SI, *S. pullorum* infection group.

To assess the intestinal barrier function across the five groups, the mRNA expression levels of *TJP1, CLDN1, CLDN2, Occludin*, and *MUC2* genes in the jejunum were analyzed by RT-qPCR. As shown in Figure 2, the expression levels of these five genes were up-regulated in the EM group relative to that in the CK group (*p* < 0.05). In contrast, the *TJP1, CLDN1, Occludin*, and *MUC2* genes were down-regulated in the SI group compared with those in the CK group (*p* < 0.05). The *CLDN1, Occludin*, and *MUC2* genes were up-regulated in the PT group relative to those in the SI group (*p* < 0.05). Additionally, all these five genes were up-regulated in the AT group compared with those in the SI group (*p* < 0.05).

### 3.5 Determination of blood biochemical indices

**Figure 3:**
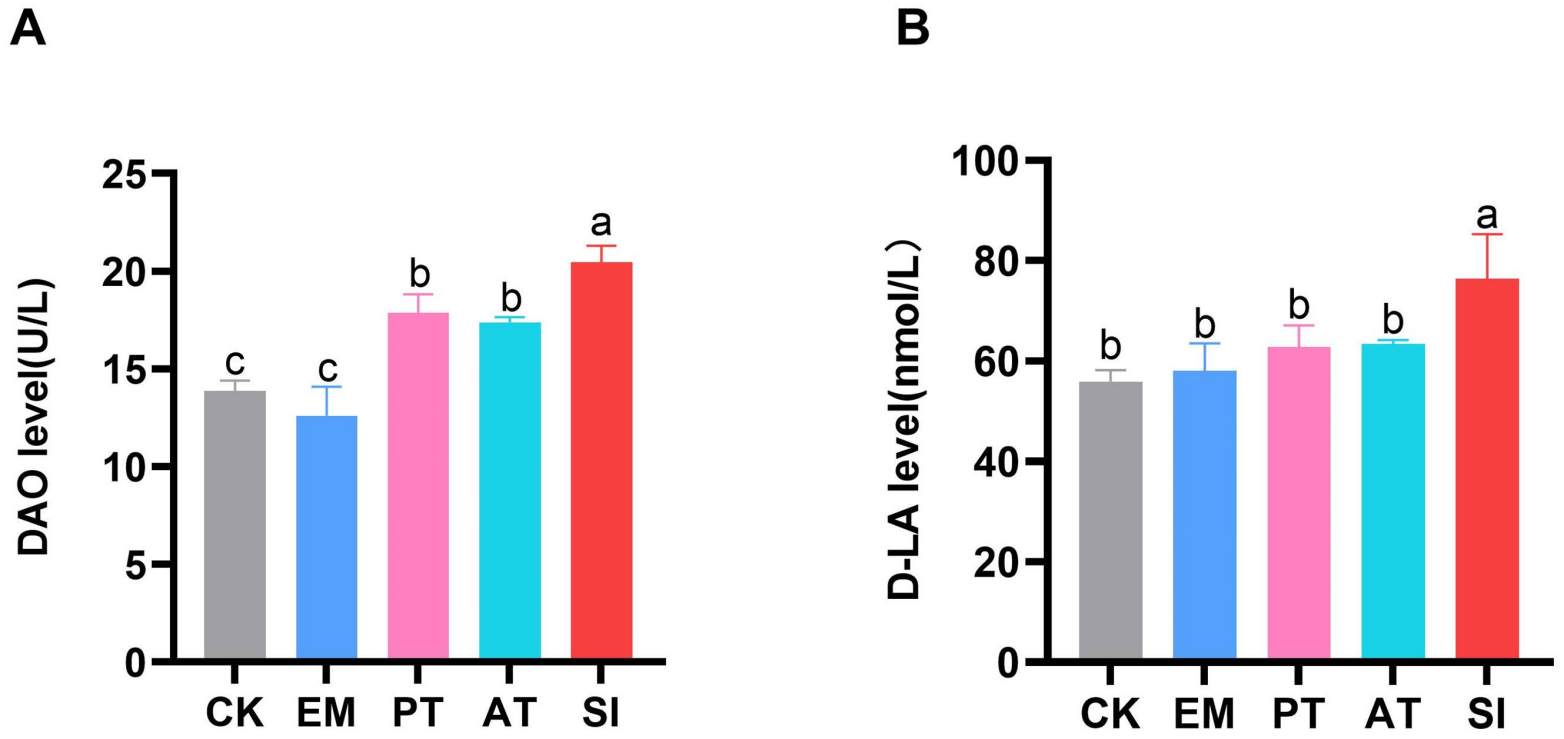
Effect of *B. coagulans* treatments on the intestinal permeability of chicks. **(A)** Serum levels of DAO and **(B)** D-LA. Differences between groups are indicated by different lowercase letters. Values are presented as the mean ± SD, *n* = 6. CK, control group; EM, *B. coagulans* group; PT, probiotics treatment group; AT, antibiotic treatment group; SI, *S. pullorum* infection group.

The levels of D-LA and DAO in serum samples were determined to assess the intestinal permeability of the five groups. As shown in Figure 3, there were no significant differences in D-LA and DAO levels between the CK and EM groups (*p* > 0.05). However, the SI group had higher DAO and D-LA levels than the CK group, which reached 20.48 U/L and 76.40 nmol/L, respectively. Additionally, the PT and AT groups showed lower DAO (17.85 U/L and 17.38 U/L) and D-LA (62.82 nmol/L and 63.34 nmol/L) than the SI group (*p* < 0.05).

### 3.6 Expression of apoptosis-related genes

**Figure 4:**
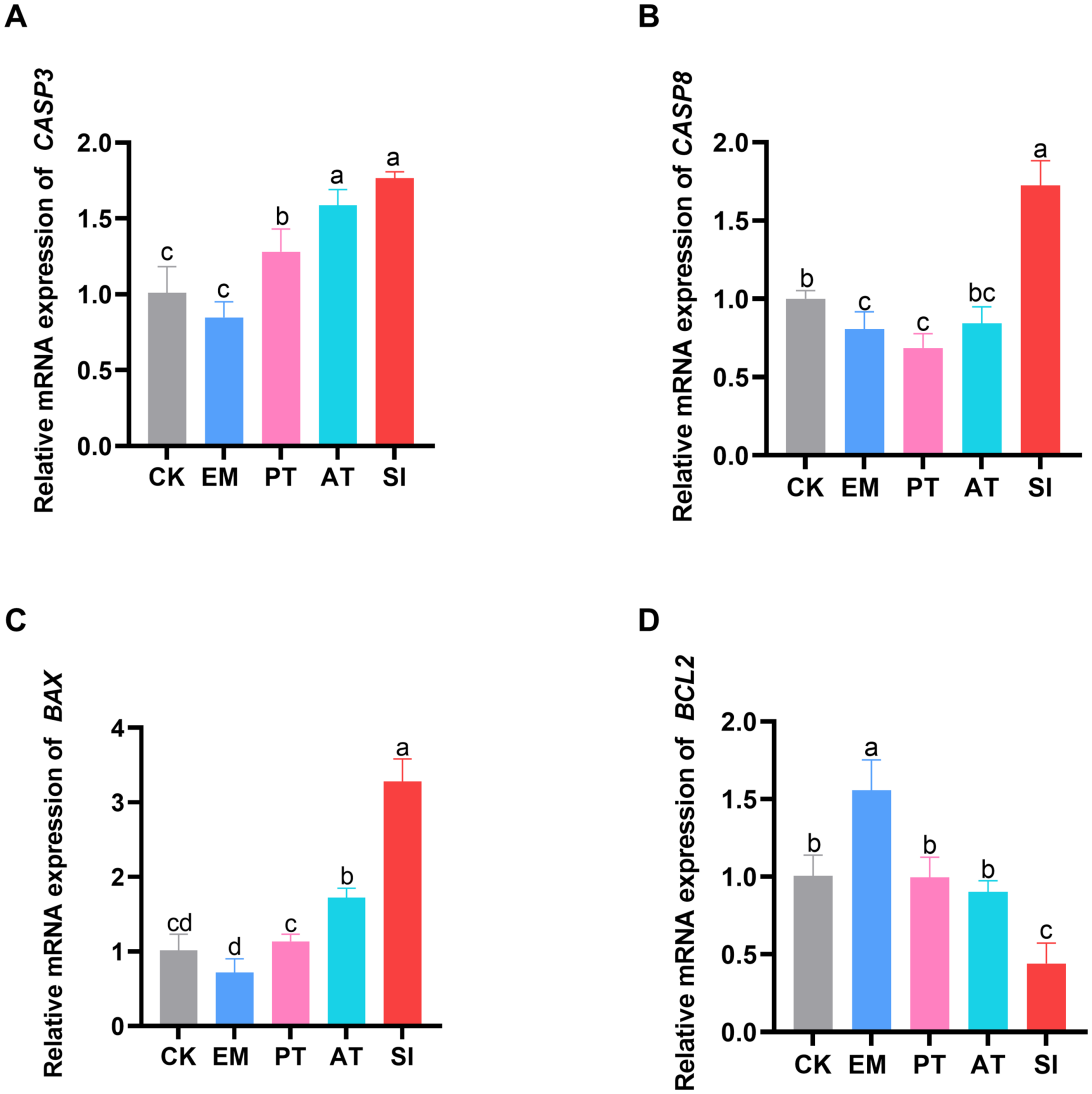
Effect of *B. coagulans* on cell apoptosis. **(A)** Relative expression of the mRNAs of *CASP3*, **(B)** *CASP8*, **(C)** *BAX*, and **(D)** *BCL2* is shown. Different lowercase letters indicate differences between groups. Values are presented as the mean ± SD, *n* = 6. CK, control group; EM, *B. coagulans* group; PT, probiotics treatment group; AT, antibiotic treatment group; SI, *S. pullorum* infection group.

The expression levels of apoptosis-related genes (*BAX, BCL2, CASP8*, and *CASP3*) in the jejunum are shown in Figure 4. The pro-apoptotic gene *CASP8* was down-regulated and the anti-apoptotic gene *BCL2* was up-regulated in the EM group compared with those in the CK group (*p* < 0.05). The expression of *BAX, CASP8*, and *CASP3* was up-regulated (*p* < 0.05), and that of *BCL2* was down-regulated in the SI group (*p* < 0.05). Moreover, *BAX, CASP8*, and *CASP3* were down-regulated (*p* < 0.05) and *BCL2* was up-regulated in the PT group compared with those in the SI group (*p* < 0.05). Compared with the SI group, the AT group showed significant down-regulation of *BAX* and *CASP8* (*p* < 0.05), while significant up-regulation of *BCL2* (*p* < 0.05).

### 3.7 Expression of proliferating cell nuclear antigen

**Figure 5:**
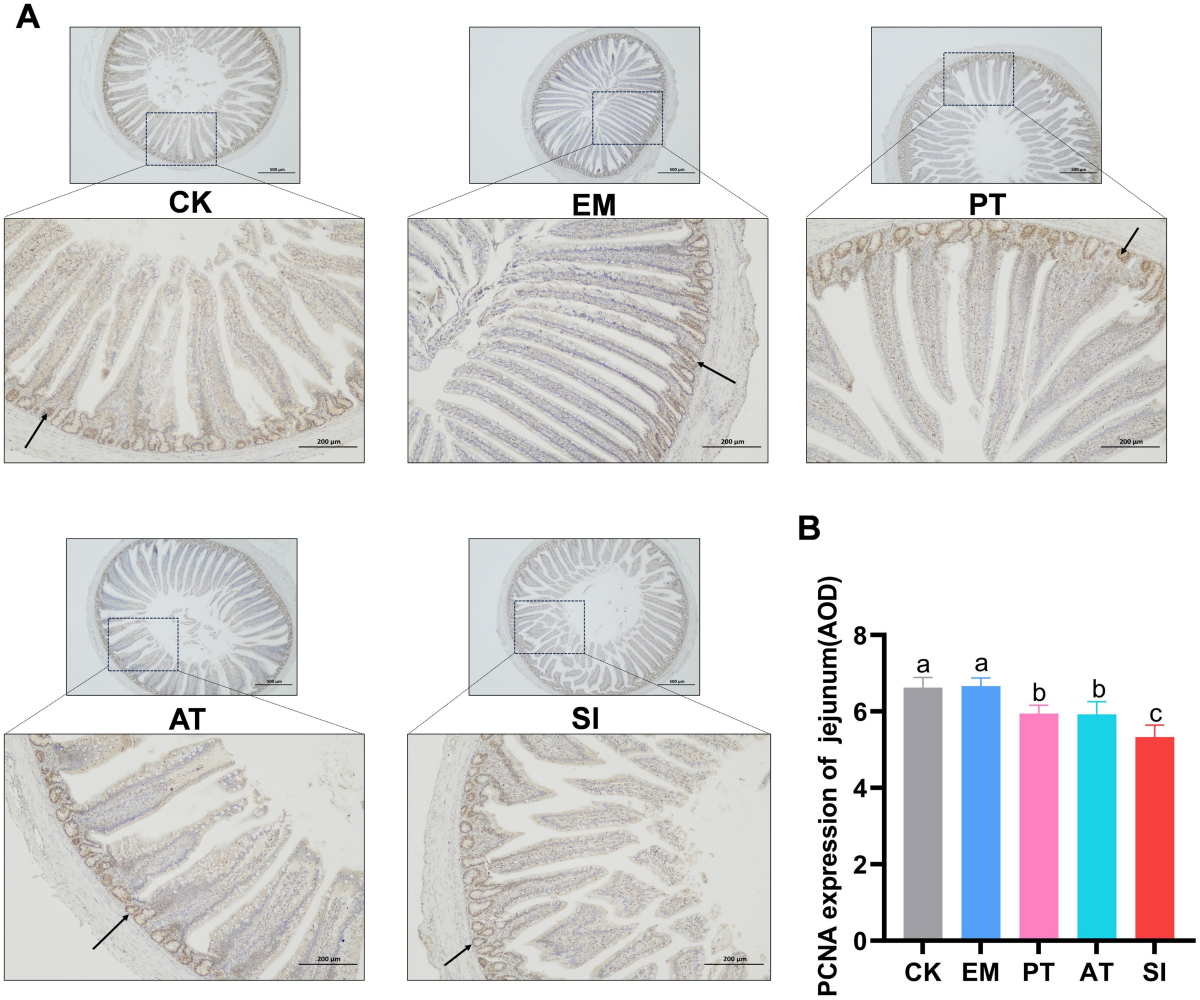
Expression of proliferating cell nuclear antigen (PCNA) in the jejunum. **(A)** PCNA was detected by immunohistochemical staining and **(B)** expression of PCNA in the jejunum (AOD) was measured. The arrow in the figure refers to a part of the stained PCNA-positive cells. Different lowercase letters indicate differences between groups. All values are presented as the mean ± SD, *n* = 6. CK, control group; EM, *B. coagulans* group; PT, probiotics treatment group; AT, antibiotic treatment group; SI, *S. pullorum* infection group.

The effect of *B. coagulans* MF-06 on expression of proliferating cell nuclear antigen (PCNA) in jejunum is shown in Figure 5. The expression of PCNA showed no significant difference between the EM group and the CK group (*p* > 0.05). In contrast, the PCNA expression in the SI group decreased (*p* < 0.05). Moreover, the PT and AT groups had higher expression of PCNA than the SI group (*p* < 0.05).

### 3.8 Expression of genes involved in the Wnt/*β*-catenin signaling pathway

**Figure 6:**
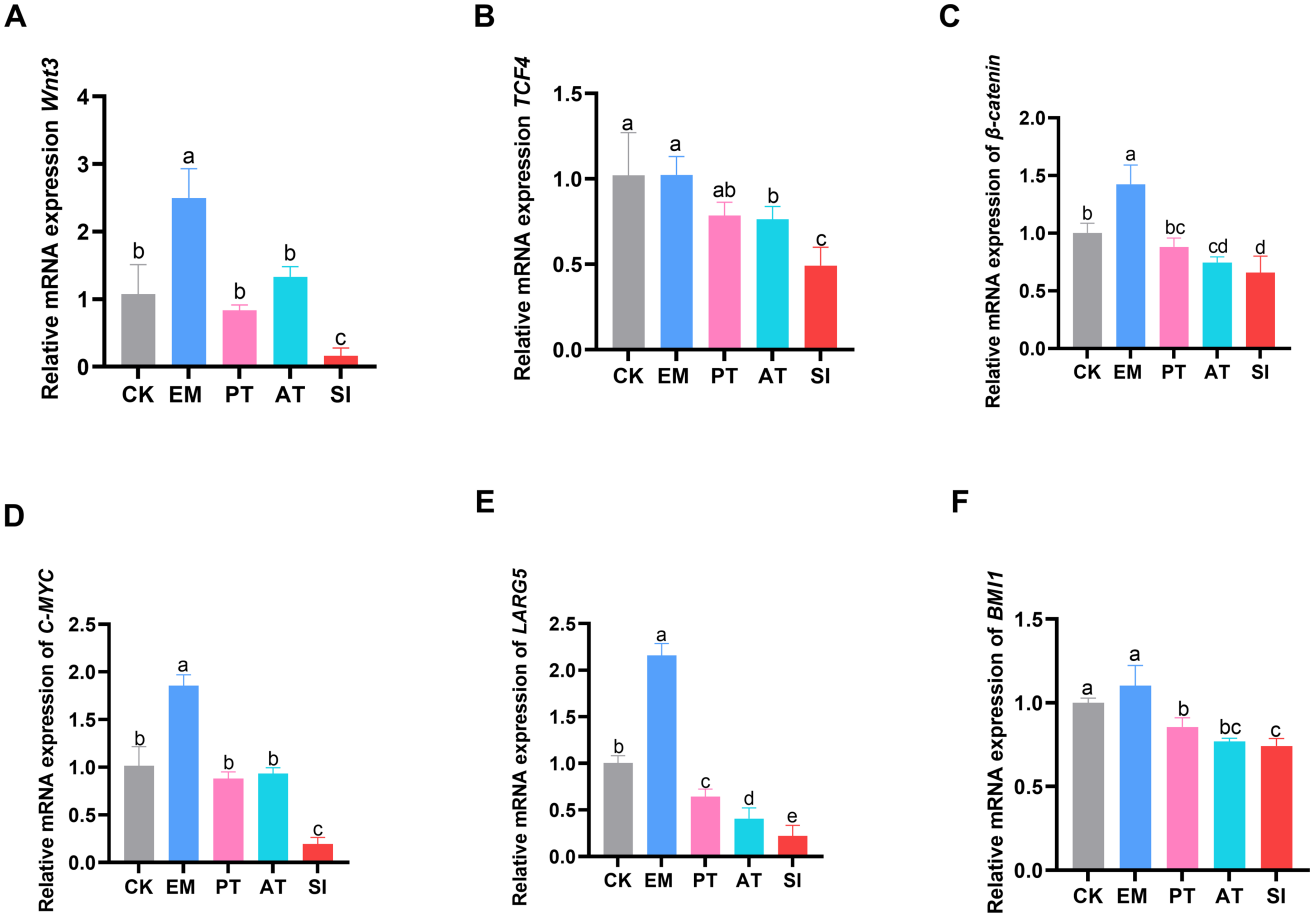
Effect of *B. coagulans* on the Wnt/β-catenin signaling pathway. **(A)** Relative expression of the mRNAs of *Wnt3*, **(B)** *TCF4*, **(C)** *β-catenin*, **(D)** *C-MYC*, **(E)** *LARG5*, and **(F)** *BMI1*. The arrow in the figure refers to a part of the stained C-MYC-positive cells. All values are presented as the mean ± SD, *n* = 6. CK, control group; EM, *B. coagulans* group; PT, probiotics treatment group; AT, antibiotic treatment group; SI, *S. pullorum* infection group.

**Figure 7:**
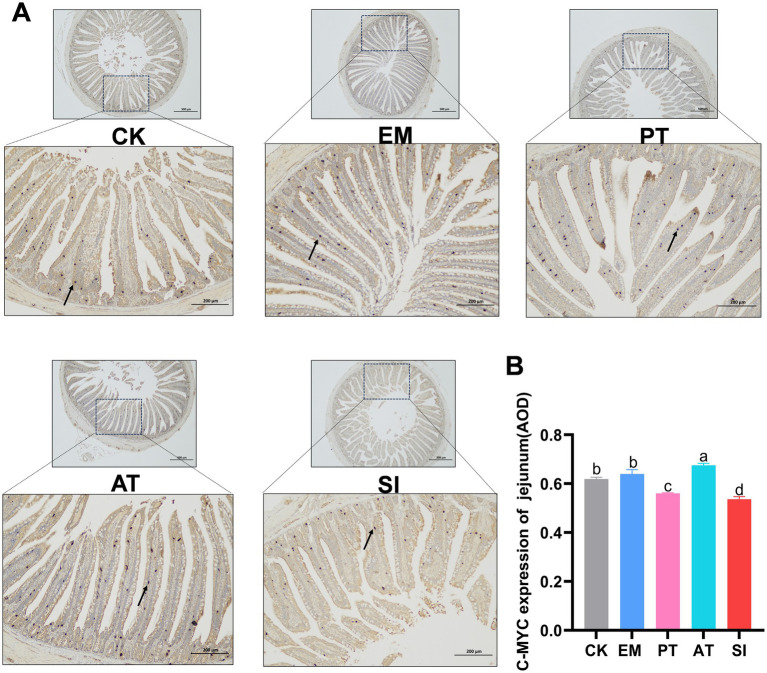
Effect of *B. coagulans* on the Wnt/β-catenin signaling pathway. **(A)** C-MYC was detected by immunohistochemical staining and **(B)** expression of C-MYC in the jejunum (AOD) was measured. Different lowercase letters indicate differences between groups. All values are presented as the mean ± SD, *n* = 6. CK, control group; EM, *B. coagulans* group; PT, probiotics treatment group; AT, antibiotic treatment group; SI, *S. pullorum* infection group.

As shown in Figure 6, The EM group had higher expression of *Wnt3, C-MYC, β-catenin*, and *LGR5* than the CK group (*p* < 0.05). In contrast, the SI group had lower expression of all the genes related to the Wnt/β-catenin signaling pathway (*p* < 0.05) compared with the CK and PT group, and lower expression of *Wnt3, TCF4, C-MYC*, and *LGR5* (*p* < 0.05), but non-significant increase in the expression of *β-catenin* and *BMI1* (*p* > 0.05) compared with the AT group. Immunohistochemistry (Figures 7A,B) showed that compared with that of the CK group, the expression of C-MYC protein in jejunum was higher (*p* < 0.05) in the EM group, but lower in the SI group (*p* < 0.05). Moreover, the PT and AT groups showed higher expression of the C-MYC protein than the SI group (*p* < 0.05).

### 3.9 Changes in gut microbiota of chicks

**Figure 8:**
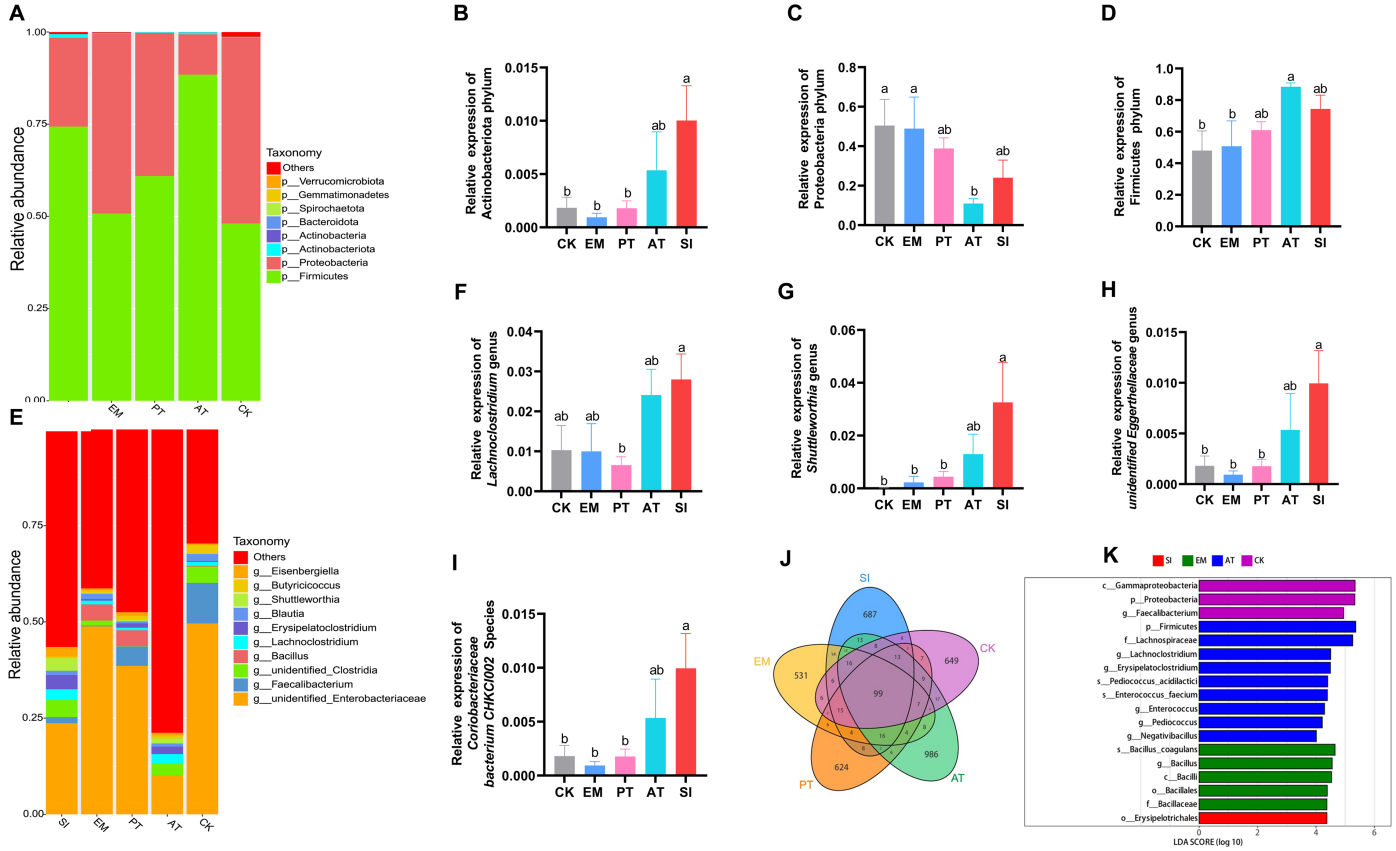
Changes in the microbial community in the chick gastrointestinal tract. **(A)** A comparison of the distribution of the top 10 bacterial communities among different treatment groups at the phylum level. Distribution of **(B)** Actinobacteriota, **(C)** Proteobacteria, and **(D)** Firmicutes in the five groups. **(E)** A comparison of the distribution of the top 10 bacterial communities among various treatment groups at the genus level. Distribution of **(F)** *Lachnoclostridium*, **(G)** *Shuttleworthia*, **(H)** *Unidentified Eggerthellaceae*, and **(I)** *Coriobacteriaceae bacterium CHKCI002* in the five groups. **(J)** Venn diagram of the distribution of ASVs in the five groups. **(K)** Differentially enriched taxa were identified in the five groups by LEfSe analysis. Different lowercase letters indicate differences between groups. All values are presented as the mean ± SD, *n* = 4. CK, control group; EM, *B. coagulans* group; PT, probiotics treatment group; AT, antibiotic treatment group; SI, *S. pullorum* infection group.

We evaluated the cecal microbial richness through 16S rRNA sequencing. The results are presented in Figure 8. The petal diagram for the structure of the gut microbiota demonstrates the common and unique ASVs across different groups: there were 99 common ASVs, and the unique ASVs included 649 ASVs in CK, 531 ASVs in EM, 624 ASVs in PT, 986 ASVs in AT, and 687 ASVs in SI (Figure 8J). The *α*-diversity, which reflects species abundance and diversity in a single sample, showed no significant difference in the Chao, Ace, and Simpson indices (*p* > 0.05), but Shannon showed a distinction trend among 5 groups (*p* = 0.06), with a significant difference between the AT group and the CK group (*p* < 0.05) (Table 5). According to β-diversity results, principal component analysis (PCA) showed a distinction trend (*p* < 0.05), which implied that the structure of intestinal microbiota was pronounced altered by *B. coagulans* MF-06 supplementation.

**Table 5:** Alpha diversity of the microorganisms in the cecum.^1^

Item	CK	EM	PT	AT	SI	*p*-value
Shannon	4.53 ± 0.57^a^	3.23 ± 1.23^ab^	3.62 ± 0.93^ab^	4.95 ± 0.56^b^	3.15 ± 1.30^ab^	0.066
Simpson	0.88 ± 0.06	0.66 ± 0.22	0.79 ± 0.10	0.92 ± 0.06	0.67 ± 0.22	0.079
Chao1	332.61 ± 66.45	257.01 ± 81.87	301.36 ± 72.27	390.39 ± 67.23	289.67 ± 94.02	0.199
ACE	359.43 ± 82.71	278.30 ± 78.64	331.87 ± 86.71	403.06 ± 62.03	314.90 ± 88.32	0.291
PD whole tree	19.36 ± 2.34	17.60 ± 6.79	17.48 ± 4.30	23.39 ± 2.26	19.04 ± 5.75	0.410

CK, control group; EM, *B. coagulans* group; PT, probiotics treatment group; AT, antibiotic treatment group; SI, *Salmonella pullorum* infection group. ^1^Values are presented as mean ± SD, *n* = 4. ^a, b, c^ Significantly different means are indicated by distinct superscripts within the same row (*p* < 0.05).

We then conducted a taxonomic analysis to compare the relative abundance at the phylum, genus, and species levels. In terms of different phyla, Actinobacteriota, Proteobacteria, and Firmicutes were predominant (Figure 8A). The EM group showed no significant differences in the relative abundance of these three phyla from the CK group (*p* > 0.05). The SI group exhibited 4.46 times more abundant Actinobacteriota than the CK group (*p* < 0.05) (Figure 8B), but no significant difference in the relative abundance of Proteobacteria and Firmicutes (*p* > 0.05) (Figures 8C,D). Compared with the SI group, the PT group exhibited a 0.82-fold decrease in the relative abundance of Actinobacteriota (*p* < 0.05) (Figure 8B), but no significant difference in Proteobacteria and Firmicutes (*p* > 0.05) (Figures 8C,D), while the AT group showed no significant differences in the relative abundance of all the three phyla (*p* > 0.05).

At the genus level, there were statistically significant differences in the relative abundance of *Lachnoclostridium, Shuttleworthia,* and *Unidentified_Eggerthellaceae* among groups (*p* < 0.05) (Figure 8E). The SI group had higher relative abundance of *Shuttleworthia* and *Unidentified_Eggerthellaceae* than the CK group and the PT group (*p* < 0.05), but no significant differences in the relative abundance of these genera from the AT groups (*p* > 0.05) (Figures 8F–H). At the species level, the SI group showed higher relative abundance of *Coriobacteriaceae bacterium CHKCI002* than the CK and PT groups (*p* < 0.05), but no significant difference from the AT group (*p* > 0.05) (Figure 8I). Based on the linear discriminant analysis (LDA), we identified 18 species with significant differences in relative abundance across all taxonomic levels (Figure 8K). The results indicated that *B. coagulans* MF-06 can maintain intestinal health and mitigate *S. pullorum*-induced inflammation by modulating cecal microbial composition.

### 3.10 Correlation analysis

**Figure 9:**
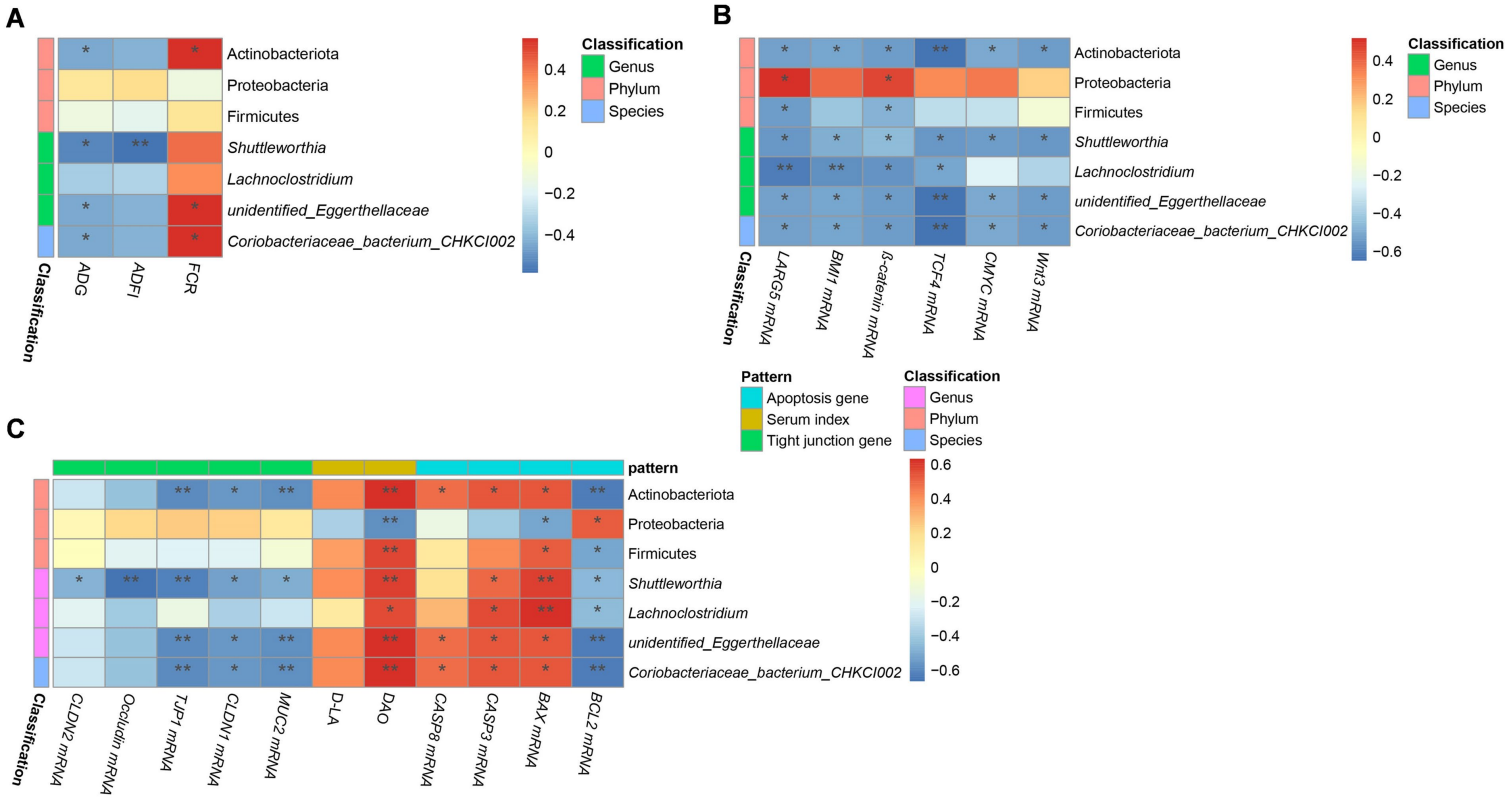
Correlation analysis of growth performance, serum permeability indicators, gene expression, and microbiome in chicks. Correlation between **(A)** performance and microbiome, **(C)** serum permeability indicators, tight junction-related gene expression, and microbiome, and **(B)** Wnt/β-catenin-related gene expression, apoptosis-related gene expression, and microbiome in the five groups. The names of microbiota are provided on the right of the graph; and the bottom presents the growth performance indices in **(A)** and the names of gene or serum indicators in **(B,C)**. The red and blue squares represent positive and negative correlations, respectively. Statistically significant correlations are represented by *p < 0.05, **p < 0.01, and ***p < 0. 001. CK, control group; EM, *B. coagulans* group; PT, probiotics treatment group; AT, antibiotic treatment group; SI, *S. pullorum* infection group.

The results of Spearman correlation analysis for microbiome and production performance, Wnt/*β*-catenin signaling pathway-related genes, and intestinal barrier indices are presented in Figures 9A–C. The relative abundance of Actinobacteriota, *Unidentified_Eggerthellaceae*, and *Coriobacteriaceae bacterium CHKCI002* was negatively correlated with ADG and the mRNA expression of *Wnt3, TCF4, C-MYC, β-catenin, BMI1, LGR5, BCL2*, *TJP1, CLDN1* and *MUC2*, but positively correlated with FCR, the mRNA expression of *BAX, CASP, CASP8*, and DAO content.

## 4 Discussion

Pullorum disease (PD) caused by *S. pullorum* is an avian-specific septicemic disease responsible for significant economic losses in the poultry industry (Barrow and Neto, 2011). Antibiotics can be used to control *S. pullorum* infection. However, prolonged use of antibiotics can lead to the emergence of antibiotic-resistant strains and disrupt the balance of the intestinal microbial ecosystem. Probiotics have been shown to maintain the stability of intestinal barrier (Liu et al., 2023). Probiotics have different types of effects and action mechanisms, such as competition with pathogenic bacteria, secretion of antimicrobial substances, and modulation of intestinal pH (Kocot et al., 2022). These probiotics are promising alternatives to antibiotics in the poultry industry (Zhou et al., 2024). However, the specific mechanisms for the effects of probiotics on the intestine of chicks infected with *S. pullorum* remain unclear.

In this study, we isolated *B. coagulans* MF-06 from the intestines of healthy adult chickens. Treatment of chicks with *B. coagulans* MF-06 alleviated symptoms such as drowsiness, rapid breathing, depressed mood, poor appetite, poor growth, and sticky white or pale-yellow feces excretion. These symptoms were similar to those reported in previous studies (Barrow and Neto, 2011; Chen et al., 2020; El-Sharkawy et al., 2020; Peng et al., 2022), confirming that *B. coagulans* MF-06 can prevent and treat infections caused by *S. pullorum* in chicks.

As *B. coagulans* MF-06 showed therapeutic effects, we evaluated the growth performance of chicks in different treatment groups. Chicks infected with *S. pullorum* showed a decrease in BW compared to the CK group. However, treatment with *B. coagulans* MF-06 and antibiotics increased BW and decreased FCR. Some studies have shown that dietary supplementation with *B. coagulans* can increase BW and ADG of broiler chicks (Fitzpatrick, 2013; Zhen et al., 2018). For example, It has been shown that infection with *Clostridium perfringens* decreased the BW (344.7 g) in broilers, whereas dietary supplementation with *B. coagulans* decreased FCR (1.74) and increased BW (377.2 g) (Wu et al., 2018). Our results are consistent with previous studies, suggesting that *B. coagulans* MF-06 supplementation improves the growth performance of chicks, probably because the bacteria secrete beneficial enzymes and growth-promoting factors (Al-Otaibi et al., 2023), which improve the digestive and absorptive capacity and reduce the colonization rate of pathogens, thus protecting the intestine (El-Moneim et al., 2020; Kai, 2021).

*S. pullorum* colonizes the intestinal wall of chicks, invades intestinal mucosal cells, and migrates through the bloodstream, causing systemic infection (Barrow and Neto, 2011). To determine the effect of *B. coagulans* on the colonization of pathogenic bacteria in chick intestine, we measured the bacterial load in the chick intestines. The results showed that *B. coagulans* MF-06 reduced the colonization of *S. pullorum* in the duodenum, jejunum, ileum, and cecum of chicks. Thus, we speculate that the *B. coagulans* MF-06 used in this study may decrease *S. pullorum* colonization in the gut, and further lower the infection rate of *S. pullorum* in chicks.

To evaluate the effect of *B. coagulans* MF-06 on *S. pullorum* colonization in chicks at the physiological level, we examined the intestinal physical barrier, including physical connection, cell proliferation, and apoptosis after *S. pullorum* infection. Intestinal epithelial cells (IECs) are important for the establishment of physical connections and preservation of the intestinal physical barrier integrity. These connections, which are involved in tight junctions (TJs), adherens junctions (AJs), and desmosomes (Buckley and Turner, 2018), can effectively prevent the leakage of substances from the intestinal lumen (Vancamelbeke and Vermeire, 2018). TJs are generally constituted by transmembrane proteins, including Claudins, occludins, peripheral membrane proteins (ZO), and regulatory proteins (Buckley and Turner, 2018). They can greatly restrict pathogen invasion from the intestinal lumen into deeper tissues, thus contributing to the defense system in the body. We found that diet supplementation with *B. coagulans* MF-06 enhanced the transcription level of *TJP1*, *CLDN1*, *CLDN2*, *MUC2*, and *Occludin* genes.

DAO, an enzyme secreted by intestinal epithelial cells, and D-LA, a metabolic product of intestinal bacteria, are released into the bloodstream when the intestinal mucosal barrier. Thus, intestinal permeability and integrity can be monitored by assessing blood levels of D-LA and DAO (Wu et al., 2022). It has been reported that *S. typhimurium* infection induced high serum DAO levels in chicks (Wang et al., 2023). Similarly, our results showed that the serum DAO and D-LA levels increased in chicks after being challenged with *S. pullorum*. Treatment of *S. pullorum*-infected chicks with *B. coagulans* MF-06 effectively reduced the D-LA and DAO levels in the serum, confirming that *B. coagulans* MF-06 can enhance the integrity of intestinal mucosal barrier.

To further elucidate the mechanisms underlying the effects of *B. coagulans* MF-06 on the intestinal barrier, we performed qRT-PCR to analyze the transcription levels of genes associated with the proliferation of the IECs. The Wnt/*β*-catenin signaling pathway facilitates the regeneration of the intestinal epithelium, particularly the proliferation of intestinal stem cells (ISCs) (Tong et al., 2023). The proliferation of IECs is regulated by β-catenin. LGR5 enhances Wnt/β-catenin signaling pathway, and BMI1 mediates the generation of proliferative ISCs (Liu and Chen, 2020; Li et al., 2022). When the Wnt/β-catenin signaling pathway is activated, active β-catenin will be translocated from the cytoplasm to the nucleus and interacts with T cell-specific factor/lymphoid enhancer factor, thereby enhancing the expression of target genes such as *C-MYC* and *CF/LEF* target genes (Zhang and Wang, 2020). In this study, there was a significant increase in the transcription levels of genes associated with the Wnt/β-catenin signaling pathway, including *Wnt3*, *β-catenin*, *TCF4*, *C-MYC*, *LGR5*, and *BMI1*. Our immunohistochemical analysis showed that the expression level of C-MYC was increased in the intestinal epithelium of *S. pullorum-*infected chicks treated with *B. coagulans* MF-06, further confirming that *B. coagulans* MF-06 inhibits *S. pullorum* infection by regulating the Wnt/β-catenin signaling pathway.

To preliminarily explore the effect of *B. coagulans* MF-06 on intestinal cell proliferation, we assessed the expression levels of intestinal PCNA, a cell proliferation marker associated with IECs and the reparative capacity of the intestinal mucosal epithelium. The results showed a significant increase in the expression of PCNA, suggesting that *B. coagulans* MF-06 may promote IECs proliferation within crypts, followed by migration along the crypt-villus axis (Sanden and Olsvik, 2009). An increase in PCNA level indicates an increase in cell division rate, which further contributes to the positive effect of *B. coagulans* MF-06 on the intestinal mucosal barrier integrity.

The apoptosis of IECs is critical for the regeneration of intestinal tissue and maintenance of normal structure. An imbalance between apoptosis and regeneration can severely impair the intestinal structure and function (Zhu et al., 2014). In eukaryotes, the BAX protein induces cell apoptosis (Westphal et al., 2011). Our qRT-PCR analysis showed increased transcription of the *BAX* gene in the intestinal tissues of *S. pullorum*-infected chicks. However, supplementation with *B. coagulans* MF-06 decreased the expression of *BAX*, indicating that *B. coagulans* MF-06 can alleviate cell apoptosis and maintain a balance between cell regeneration and apoptosis.

Microbiota is an integral part of the gut with important roles in nutrition, physiology, and intestinal morphology. Moreover, it is involved in the host immune defense against pathogens (Sugiharto and Ranjitkar, 2019). Some studies have shown that gut microbiota may influence the proliferation of epithelial cells. Here, we assessed the composition of the gut microbiota by high-throughput 16S rRNA sequencing. The results showed that *S. pullorum* increased the ASV number and altered the community structure. However, although *B. coagulans* MF-06 influenced the cecal microbiota structure, it led to no significant change in the *α*-diversity of bacterial community. At the phylum level, *S. pullorum* infection increased the relative abundance of Actinobacteriota in the cecum. Actinobacteriota is reported to improve feed utilization by producing extracellular enzymes, and to decompose undigested components in feed through secreting endogenous enzymes (Lee et al., 2014). In addition, Actinobacteriota is helpful for the maintenance of overall microbial structure. Due to the production of bacteriocins and the ability to convert feed into fermentable microbial biomass (Samson et al., 2020). Studies have shown that intestinal injury can result in an increased content of ethanolamine, and all kinds of non-pathogenic and pathogenic Actinobacteriota use ethanolamine as a source of carbon or nitrogen (Zeng et al., 2017). In this study, the abundance of Actinobacteriota increased in the SI group, suggesting inflammation and injury in the intestine. The effects of intestinal injury, Actinobacteriota, and ethanolamine need to be further studied. However, the PT group showed a less dramatic increase in the abundance of Actinobacteriota in the intestine than the SI group, the reduction of Actinobacteriota may be related to the fact that *B. coagulans* can produce core metabolites such as organic acids and amino acids, promote the metabolism and growth of beneficial bacteria in the cecum, reduce intestinal pH value, prevent the growth and proliferation of pathogenic bacteria, regulate the balance of intestinal flora, improve the intestinal environment, and maintain the integrity of intestinal epithelium (Li et al., 2021; Xiong et al., 2024). Some studies have found that heat stress impairs the antioxidant system and intestinal barrier function, resulting in a significant increase in Actinobacteria at the phylum level in the gut of broilers, while dietary Ellagic Acid significantly reduces their abundance (Yang et al., 2022), which is similar to the results of the present study. In addition, our results showed that the relative abundances of *Lachnoclostridium, Shuttleworthia*, and *unidentified_Eggerthellaceae* were dramatically increased in SI groups compared to PT group at the genus level. *unidentified_Eggerthellaceae* is a bacterial group that is positively correlated with inflammation, may promote intestinal inflammation, and is widely involved in the etiology of gastrointestinal diseases (Aparicio et al., 2020; Xia et al., 2023). Therefore, the addition of *B. coagulans* to the diet may cause competitive exclusion with pathogenic bacteria, reduce the colonization of pathogenic bacteria, and have a positive effect on intestinal health. Surprisingly, some studies have found that species of *Lachnoclostridium* and *Shuttleworthia* has produce butyrate with anti-inflammatory properties and enhance the intestinal barrier by upregulating the tight junction protein (Dai et al., 2023), which was controversial with our study. The possible reason may be due to the activation of the host immune system by *S. pullorum* infection, triggering an immune response. Flora with antioxidant or antimicrobial capabilities (including *Lachnoclostridium*) may gain a proliferative advantage to help suppress excessive growth of harmful bacteria.

Our correlation analysis showed that *Actinobacteriota*, *Shuttleworthia*, and *unidentified*_*Eggerthellaceae* had positive correlation with the DAO content in serum, but were negatively correlated with ADG, tight junction genes, and genes related to the Wnt/*β*-catenin signaling pathway. DAO, ADG, tight junction genes, and the Wnt/β-catenin signaling pathway are important indicators to evaluate the integrity, function, and health of the intestine. Therefore, we speculate that Actinobacteriota, *Shuttleworthia* and *unidentified_Eggerthellaceae* may have certain effects on the jejunal mucosa, but the specific mechanisms need to be further studied.

In summary, we elucidated the effects of probiotics on growth performance, intestinal mucosal barrier and gut microbiota of chicks challenged with *S. pullorum*. Our results demonstrated that probiotics are promising alternatives to address some challenges associated with the use of antibiotics in the poultry industry. However, this study also has some limitations. For instance, although we found that *B. coagulans* MF-06 can restrain *S. pullorum* colonization, improve the protective function of the intestinal mucosal barrier, and modulate the microbial community to prevent *S. pullorum* infection, the relevant metabolites produced by *B. coagulans* MF-06 need to be further investigated.

## 5 Conclusion

In conclusion, this study showed that avian autochthonous *B. coagulation* MF-06 can resist *S. pullorum* infection, mainly through the activation of the Wnt/β-catenin signaling pathways, regulation of apoptosis cycle, improvement of intestinal mucosal barrier and regulation of microbiota. A combined analysis of growth performance, intestinal integrity, and microbiota demonstrated the probiotic effect of *B. coagulation* MF-06. Our results will help to understand the protective mechanism of *B. coagulation* MF-06 against *S. pullorum* infection, and develop new alternative candidate strains or treatments for traditional antibiotic therapies. In addition, our study has some limitations, the present study only observed the effects of *B. coagulans* within 14 days, and the specific core metabolites of probiotics and their long-term effects on gut microbiota are still unclear and need to be further studied. In view of the findings in this study, future metabolomics studies or trials in larger animal populations will be conducted to provide more comprehensive information about probiotics on animal gut health.

## Data Availability

The data presented in the study are deposited in the NCBI repository (https://www.ncbi.nlm.nih.gov/), accession number PRJNA1150955.

## Footnotes

^1^https://github.com/OpenGene/fastp

^2^http://ccb.jhu.edu/software/FLASH/

^3^https://www.arb-silva.de/

^4^http://www.drive5.com/usearch/manual/uchime_algo.html

^5^http://drive5.com/uparse/

^6^http://www.arb-silva.de/
