# Supplementary material for: Bacillus coagulans MF-06 alleviates intestinal mucosal barrier from damage in chicks infected with Salmonella pullorum via activating the Wnt/β-catenin pathway
Source: Front Microbiol. 2024 Nov 29;15:1492035. doi: 10.3389/fmicb.2024.1492035 (PMC11638242; doi:10.3389/fmicb.2024.1492035)
Supplement: Supplementary file 1 [file Data_Sheet_1.PDF]

Supplementary Material

1 Supplementary Figure

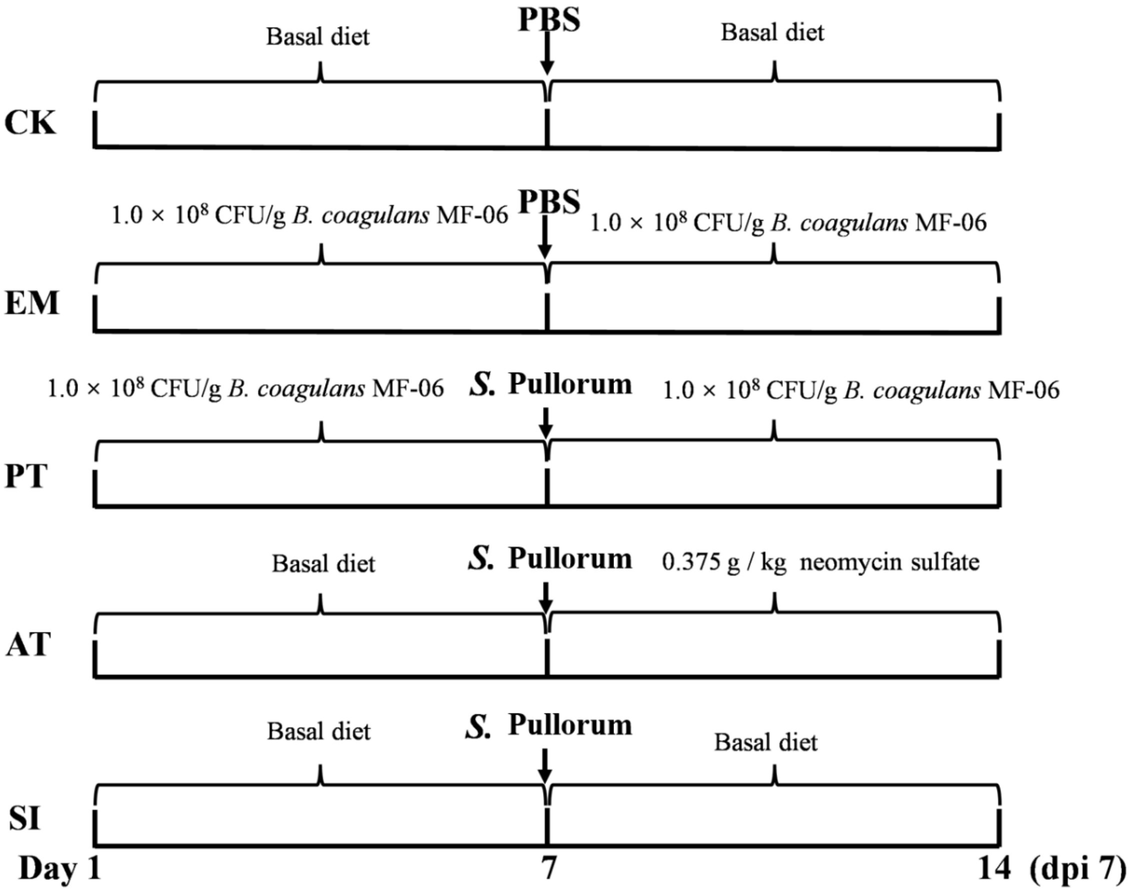

Figure 1. A total of 150 one-day-old SPF chicks (Jinghong No.1) were selected and randomly divided into five groups: control group (CK), probiotics group (EM), probiotics treatment group (PT), antibiotic treatment group (AT), *S.* pullorum infection group (SI), Each group comprised six replicates, with each replicate of five chicks. CK, AT and SI groups were fed a basal diet, EM and PT groups were fed a basal diet supplemented with 1.0×10^8 CFU/g *B. coagulans*. PT, AT and SI groups were gavaged with 1.0×10^9 CFU/0.5 mL *S.* pullorum at 7 days of age, while the chicks in CK and EM groups were gavaged with the same dose of sterile water. The chicks in AT group were fed with 0.375 g/kg neomycin sulfate in the basal diet from days 7-14.

2 Supplementary Tables

Table1. Logarithmic colony counts of *S. pullorum* in the duodenum, jejunum, ileum, and cecum of various treatment groups.[1]

Item	PT	AT	SI	P-value
Duodenum (cm)	6.21 ± 0.01[b]	6.15 ± 0.03[c]	6.68 ± 0.02[a]	<0.001
Jejunum (cm)	4.80 ± 0.05[c]	5.71 ± 0.07[b]	6.10 ± 0.10[a]	<0.001
Ileum (cm)	5.80 ± 0.05[c]	6.42 ± 0.13[b]	7.07 ± 0.57[a]	<0.001
cecum (cm)	6.43 ± 0.03[b]	6.34 ± 0.03[c]	6.99 ±0.03[a]	<0.001

Note: Abbreviations: CK, control group; EM, *B. coagulans* group; PT, probiotics treatment group; AT, antibiotic treatment group; SI, *S. pullorum* infection group

[1]Values are presented as mean ± SD, n=6.

[a, b, c] Significantly different means are indicated by distinct superscripts within the same row ($P < 0.05$).

Table 2. Effect of *B. coagulans* treatments on the intestinal permeability of chicks (Serum levels of DAO and D-LA).[1]

Item	CK	EM	PT	AT	SI	P-value
DAO (U/L)	13.89 ± 0.52^{c}	12.62 ± 1.48^{c}	17.86 ± 0.96^{b}	17.38 ± 0.27^{b}	20.48 ± 0.83^{a}	<0.001
D-LA (nmol/L)	55.85 ± 3.14^{b}	58.00 ± 5.27^{b}	62.82 ± 4.29^{b}	63.34 ± 0.88^{b}	76.40 ± 8.93^{a}	<0.001

Note: Abbreviations: CK, control group; EM, *B. coagulans* group; PT, probiotics treatment group; AT, antibiotic treatment group; SI, *S. pullorum* infection group

[1]Values are presented as mean ± SD, n=6.

[a, b, c] Significantly different means are indicated by distinct superscripts within the same row ($P < 0.05$).

Table 3. Expression of proliferating cell nuclear antigen (PCNA) and v-myc avian myelocytomatosis viral oncogene homolog (C-MYC) in the jejunum.[1]

Item	CK	EM	PT	AT	SI	P-value
PCNA (%)	6.626 ± 0.266^{a}	6.663 ± 0.214^{a}	5.942 ± 0.221^{b}	5.921 ± 0.335^{b}	5.328 ± 0.317^{c}	<0.001
C-MYC (%)	0.620 ± 0.007^{b}	0.640 ± 0.017^{a}	0.565 ± 0.009^{c}	0.575 ± 0.008^{c}	0.537 ± 0.010^{d}	<0.001

Note: Abbreviations: CK, control group; EM, *B. coagulans* group; PT, probiotics treatment group; AT, antibiotic treatment group; SI, *S. pullorum* infection group

[1]Values are presented as mean ± SD, n=6.

[a, b, c] Significantly different means are indicated by distinct superscripts within the same row ($P < 0.05$).
